# 1713. B-Cell Subsets in Children and Adolescents Living with Perinatally-Acquired HIV

**DOI:** 10.1093/ofid/ofad500.1546

**Published:** 2023-11-27

**Authors:** German Contreras, ramia g zakhour, Gloria Heresi, James Murphy

**Affiliations:** University of Texas McGovern Medical School, houston, Texas; University of Texas at Houston, houston, Texas; UTHealth, McGovern Medical School, Houston, Texas; UTHealth, McGovern Medical School, Houston, Texas

## Abstract

**Background:**

To gain insight into the mechanisms of immune deficits persisting in children and adolescents living with perinatally-acquired HIV (PHIV) on ART at 4, 6, and 10 years.

**Figure:**
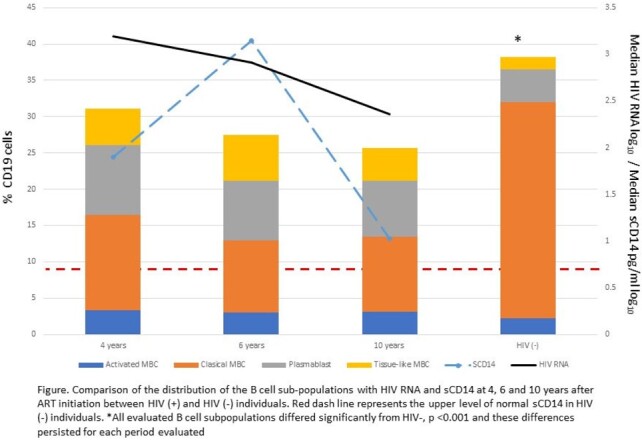

**Methods:**

We determined four B-cell subpopulations by flow cytometry after initiation of ART -classical, plasma blast activated, and tissue-like memory B cell MBCs. Macrophage activation was assessed by measuring plasma levels of sCD14. Baseline characteristics and clinical data of study participants were extracted from medical records. Categorical and continuous variables were compared by the Fisher or Wilcoxon rank-sum test.

**Results:**

Eleven PHIV and 22 HIV- were studied. The median age at ART initiation was 4.5 (interquartile range [IQR], 3.2–12.4) years, median CD4% was 23.4 (IQR, 18–31), and plasma median HIV RNA was 4.3 (IQR, 3.1–5.2) log_10_ copies/mL. Median CD4% and plasma HIV RNA log_10_ copies/mL at 4, 6, and 10 years were 28 and 3.2; 27.9 and 2.9; and 29.5 and 2.4.

HIV (+) compared to HIV (-) had significantly lower percentages of classical MBCs but higher percentages of plasmablast, activated, and tissue-like MBCs (Figure). Moreover, these differences persisted through 10 years (Figure). Of note, the distribution of the B-cell subpopulations did not change during the six years of evaluation for the PHIV, despite progressive control of HIV replication and immune activation measured as sCD14. (Figure).

**Conclusion:**

Children and adolescents living with perinatally acquired HIV had persistent abnormal B-cell subpopulation distribution despite an effective HIV viremia control and CD4 % restoration following ART initiation. The linkage between the observed persistent immune activation and abnormalities observed within the B cell compartment deserves investigation. The abnormalities in B-cell subpopulations might explain our population's low immune response to vaccines.

**Disclosures:**

**All Authors**: No reported disclosures

